# Personal Perspectives: Having a Prostatectomy and the Role of the Cancer Specialist Nurse

**DOI:** 10.2147/IJGM.S267559

**Published:** 2020-10-19

**Authors:** Simon D Taylor-Robinson, Kathy Dykes, Bethan Hawkes

**Affiliations:** 1 Department of Surgery and Cancer, Imperial College London, St Mary’s Hospital Campus, London W2 1NY, UK; 2 Department of Surgery, Morriston Hospital, Swansea, Wales SA6 6NL, UK; 3 Wales Cancer Network, NHS Wales Health Collaborative, Cardiff, Wales CF15 9SS, UK

**Keywords:** prostatectomy, specialist cancer nursing, psychological complications, physical complications

## Abstract

**Background:**

Doctors are often ill-prepared to become patients, despite knowing the technicalities of surgical procedures and the day-to-day workings of hospital life intimately. Surrendering the decision-making process to other healthcare professionals can be an unnerving process for many of those who are medically qualified.

**Aim:**

Although the sequelae of prostatectomy have often been written about, little is in the literature from medically qualified patients about their personal experiences of the procedure. We aimed to highlight areas where communication between medically qualified patients and their carers may be strengthened.

**Methods and Results:**

We present a personal perspective of the emotional issues surrounding a potential cancer diagnosis, the experience of having a prostatectomy and what the hospital encounters were like in reality with a viewpoint of informing the medical profession in providing better patient information when they ask “what will it be like?”. From this perspective, the critical role of the cancer specialist nurse is highlighted as the lynch pin in providing a continuing source of information to medically qualified patients and in not treating them as omniscient, simply because of a medical degree.

**Conclusion:**

Prostatectomy is a common procedure, but often questions about recovery after the procedure including impotence and incontinence are left unanswered in dealing with medically qualified colleagues when they are patients. Human behaviour is predictable, and medically qualified patients are just as apt to forget what is said to them as anyone else. However, the central role of the cancer specialist nurse as the bridge between the medical team and the patient should not be underestimated.

## Introduction

In the United Kingdom, it is not a mandatory screening policy to have an annual serum prostate-specific antigen (PSA) measurement performed in men over the age of 50 years old.^1^

Prostatectomy for benign or malignant causes is not a procedure that should be undertaken lightly, as it has significant potential side-effects which are both physical and psychological. The list of problems is long but includes pain, haematuria, clot retention, peri- or post-operative cardiac sequelae in the elderly, impotence, absence of ejaculation and incontinence to name but a few physical issues that confront many men.^2^

The psychological issues that accompany these problems are both short and longer term in nature.^2^ Fear in many men undergoing a prostatectomy means that the impact of the diagnosis may not be fully understood and even if told, potentially longer-term issues such as incontinence, impotence and lack of ejaculate may not be fully grasped in the pre-operative period, where there may be a whirl of investigations, biopsies, MRI scans and appointments to keep.

With time, depression can fill the recovery period.^3^ Thus, many men find it difficult to adjust, even when the operation has been a success.

We present a personal perspective of having a prostatectomy from a medically qualified patient and the critical role of the cancer specialist nurse (CSN; KD) as a liaison person between patient and doctor, but also as a sounding board for patient fears and worries, both in the pre-operative period and during recovery.

## Methods

This personal perspective on prostatectomy was drawn from direct patient discussions and the daily diary of a medically qualified patient in the pre-operative and peri-operative period from February to May 2019. The patient was known to all three authors. Written, informed consent was obtained from the patient for publication of the diary extracts and for transcription of the conversations. The sections on the role of the CSN were drawn from personal experience of the second author (KD) and from feedback provided from patient satisfaction questionnaires, completed in 2019 by patients treated in South Wales, where the author works.

## Results

### Personal Perspectives – Prostatectomy

#### Pre-Operative Period

The medically qualified patient went voluntarily to his family practitioner from the age of 55 years for a 12-monthly PSA check. Like many men in their late 50s, he had some slowing of his urinary flow with a stop-start action and post-micturition dribbling. He reported that he thought very little of it until one day in late January 2019, his doctor said to him that his PSA had doubled since the previous year. A MRI of the prostate was advised and he duly went to the prostate cancer screening clinic in his local hospital, not believing that anything would be amiss. The patient reported that the junior doctor whom he saw insisted upon a digital rectal examination, despite having the results of the MRI. He then reported that the junior urologist told him that there was a suspicious lesion on the scan and that she needed to speak to her consultant. The patient recalled that the junior urologist spoke in a matter of fact sort of way, as if speaking about another patient to a medical colleague. The consultant then arrived, explained that the patient would need multiple prostatic biopsies and then left. Although medically qualified, the patient had to ask the nurse how this was performed (via a transperineal route as it turned out), as he was too shocked to ask the young doctor. All he remembered was that he would be taken in for biopsy the following week. He maintained that he was given no information on what to expect and went home to read Stephen Fry’s experiences of his prostatectomy as the nearest form of comfort.^4^

#### The Prostatic Biopsy

Perhaps because he was a doctor, the patient considered that the team assumed knowledge on his part and that therefore the informed consent for the multiple prostatic biopsies the following week appeared at best perfunctory. The patient recounted that the middle-grade doctor effectively said “just sign here”, reassuring him that he must be familiar with everything. He recalled that he much preferred the anaesthetist whose patronising tones at least left no stone unturned with his pre-set patter meted out to everyone in the day ward in turn about anaesthetic risks and although medically qualified, the patient was no exception to the anaesthetist’s heavily time-rehearsed speech.

Nobody had mentioned the possibility of going into urinary retention post-biopsy, but into retention he rapidly went. He recalled that the junior doctor forgot to use anaesthetic gel when catheterising him and when he winced, she said “haven’t you been catheterised before?” in sharp tones. He reported in his diaries that he felt at sea mentally, but that he was determined to be a good patient and was super-nice to the nurses and the healthcare assistants, trying to crack jokes to cover his fear and uncertainty. He was discharged with the wrong catheter bags (without a tap at the bottom) with an appointment the following week for a trial without catheter. He reported in his diaries, the following:

To say I bled alarmingly with lots of ‘blackcurrant jelly’-style clots was an understatement, but nobody prepared me for that either. I sat at home and worried.

#### Catheter Removal

The following week, he went for the catheter removal which was done efficiently, but after 4 jugs of water and several cups of tea, he knew he was in retention again. A portable ultrasound was used to assess his bladder size and he was quickly informed that he was not in retention and that he had to drink more water. He then reported that when he was in extremis, he called again for help, having spent the morning on the toilet in the futile effort to pass urine. He was again told that the ultrasound showed that he did not have a bladder. The patient palpated it himself and said “have you ever thought that your test may be wrong?” At that point, the specialist cancer nurse arrived, assessed the situation and promptly catheterised him, relieving the patient of several litres of still-blood-stained urine. The patient reported in his diaries that the specialist cancer nurse was kind, reassuring and gave the patient both time and the opportunity to ask questions which he had not voiced previously, either because the medical staff involved had assumed knowledge on the patient’s part or because they were too busy. The literature given to him and the knowledge that he had a friendly person to phone if he needed it was immensely reassuring.

#### Peri-operative Period

When the day of the prostatectomy dawned, nobody talked about the possibility of incontinence post-procedure (probably because of assumed knowledge on the medically qualified patient’s part) and the doctor taking consent made light of the possibility of impotence by saying “I daresay, you won’t be needing that anymore, at your age”. The patient remembered smiling weakly, but felt in the dark as to what he would experience in the post-operative period.

When he woke from the anaesthetic he was aware of the irrigation catheter, which seemed oddly comforting, but repeated bouts of clot retention over the next 48 hours soon put things in a different and much more distressing light. He became aware that the brain is incapable of saying “my prostate hurts” and instead the feeling of intense proctalgia supervenes. He worked out that non-steroidal anti-inflammatory agents were helpful, as opiates made him constipated and made matters worse – all trial and error on the patient’s part. However, the friendly specialist nurse confirmed these suppositions and acted as a confidante when the patient felt low. Catheter removal was traumatic and the patient found it difficult to urinate owing to the passage of large clots over the ensuing few days at home. While the consultant was on holiday, the specialist nurse was able to reassure him that he would be fine and that to spend time meditating on the toilet until the clots passed would be helpful.

His post-operative period was complicated by incontinence and persistent passage of clots, but the patient reported that he felt supported by the specialist nurse who always had time for any question and was a fount of knowledge and reassurance.

The patient recalled in his diaries and by direct conversation that he remains very grateful to the surgeons, anaesthetists, theatre nurses, ward staff and the entire multidisciplinary team for an excellent job done with supreme professionalism. It should be noted that the perspective above was written not by way of criticism, but more to highlight issues in communication which are sometimes neglected, but which can be addressed to make the patient pathway smoother for future patients.

### Personal Perspectives – The Role of the CSN

The CSN is the member of the multidisciplinary team who predominantly acts as a key worker for people affected by cancer and is responsible for the delivery of person-centred care.^5^

A cancer key worker is

a person who, with the patient’s consent and agreement, takes a key role in coordinating the patient’s care and promoting continuity, ensuring the patient knows who to access for information and advice^6^

Through undertaking a holistic needs assessment, a plan of care can be developed that is centred around the person’s physical, emotional, practical, spiritual, psychological and social wellbeing.^7^ The new cancer optimal pathways in Wales describe where patients should receive consistent information and support, tailored to meet their needs.

As a CSN who works at a regional Surgery Centre, I (KD) see my role as the central “go-between” for patients referred from various health boards around the region. When considering the many professionals who are involved prior to a patient receiving their diagnosis, it is no wonder that most people do not know who to ask about what and even if told verbally or given an advice leaflet, they remain confused and afraid. The CSN is the one person whom patients can contact with questions, fears and worries and just to talk without considering that they are wasting a healthcare professional’s time.^7^ If properly introduced at the right part of the patient journey, the CSN can allay unfounded worries and explain potential, but real risks so that complications of surgery are not a surprise.

An early introduction to the CSN should be a mandatory part of the patient care pathway.^6^ I always tell patients to leave the scheduling of outstanding pre-operative tests and their results to me, as I feel I can smooth out the process for many people who culturally have silent worries and may not be able to express these easily. In so doing, I feel that trust is gained and this allows most patients to open up about “what will it be like?” The good CSN acts as the patient advocate amongst the myriad of specialists they will encounter during their treatment journey.

The CSN role “as I see it” is to be the one constant reference point for patients and their families and loved ones. If I do not know the answer to a question, I will find out. In fact, I always tell patients that no healthcare professional can hide from or avoid me when I have patient questions to answer. Patients are given my contact details (direct line with answer phone, pager and email address) at their first clinic appointment. All verbal information is backed up with written information. Patients are also directed to reliable websites that previous patients have found helpful. There are also two of us, so despite unavoidable annual leave, a CSN is available for patient support at any point. In our unit, we conduct a yearly CSN patient satisfaction survey and the suggestions given by previous patients have certainly helped shaped and improve our practice.

The CSN should treat patients as individuals regardless of their background – even medically qualified patients need the same information as the next person. This I have learned from personal experience having lost both parents to cancer. Being a CSN meant nothing when I learned they had cancer, as my mind went blank. I was not a nurse in this situation; I was simply a very scared daughter. All health professionals learn from what went well and not so well in a patient journey both personally and professionally - and I am no exception.

## Discussion

The physical and psychological aspects of prostatectomy should not be underestimated, given that these urological problems are not uncommon and affect considerable numbers of men, many of whom have decades of busy lives ahead of them.^8^ The experiences that we have described are common.^8^,^9^

To echo the issues we have discussed in this article, a metasynthesis of 15 studies (using meta-data-analysis, meta-method, and meta-theory) demonstrated how common physical, psychological, and social problems were after prostatectomy.^9^ The collated message from this meta-study was that those undergoing prostatectomy were both physically and psychologically poorly prepared for the consequences of the procedure and that is was common to have a paucity of information and support. The authors recommended that the medical profession needs to be more alert to the provision of comprehensive patient-centered informational support as a critical component of the post-prostatectomy patient journey.^9^

Furthermore, a recently published study with a multinational authorship assessed social support for patients after radical prostatectomy.^10^ Fernández-Sola and colleagues found that patient social and professional support mitigated the common medical deficiencies in terms of lack of medical information and counseling during the peri-operative and post-operative period. Much as we have outlined in our article, Fernández-Sola and colleagues showed that informational patient support needs to be stronger for those undergoing prostatectomy.^10^

A recent Canadian study highlighted that there are challenges in returning to work after prostatectomy and these are usually underestimated by both the medical team involved and by the patients themselves.^11^ With this in mind, the authors felt that further focused information in the pre-operative period might reduce anxiety levels for those undergoing the process of prostatectomy.^11^

The CSN can play a critical role in patient education through ascertaining what has been understood and what needs further explanation. Understanding unfounded worries and not skating over assumed knowledge draws on the clinical experience of the CSN, but ultimately the role is to act as the patient friend and advocate at a very difficult physical and emotional time in life.

What needs to be deduced from this article is that medically qualified patients are no different to any other and undergo the same psychological issues as anyone else undergoing a prostatectomy. No information should be withheld because of the perception that a medically qualified patient must already know the consequences of the procedure. Information should be exchanged carefully with time for reflection and for the opportunity to ask questions, no matter who the patient may be.

## Footnote

KD is a trained CSN with a specialist interest in hepatobiliary disease. STR is a hepatologist. BH is the acting manager of the Wales Cancer Network.
